# Refracture of Bones

**Published:** 1871-02

**Authors:** 


Refracture of Bones.—Dr. Skey, of Bartholomew's Hospital (*Lancet*) does not hesitate to refracture the bones in cases of deformity or shortening from imperfect dressing in the first instance. He finds no difficulty in doing so, and no risk of breaking the bone elsewhere than at the proper point. The femur he has broken at the expiration of eleven weeks, and the bones of the forearm in seventeen weeks after the original fracture. In the femur, when great force is required, he places the limb on the edge of a table, which is covered by several folds of blanket, and brings the weight of his body to bear gradually on it—anæsthesia having been first produced.—*Pacific Medical and Surgical Journal.*

